# The role of voltage-gated calcium channel α2δ-1 in the occurrence and development in myofascial orofacial pain

**DOI:** 10.1186/s12903-024-04338-y

**Published:** 2024-05-12

**Authors:** Yang Lu, Jingfu Wang, Li Li, Xiaodong Zhang

**Affiliations:** Department of Stomatology, General Hospital of Northern Theater Command, No.83, Wenhua Road, Shenhe District, Shenyang, 110016 China

**Keywords:** Voltage-gated calcium channel, Myofascial orofacial pain, Cavα2δ-1

## Abstract

Patients who suffer from myofascial orofacial pain could affect their quality of life deeply. The pathogenesis of pain is still unclear. Our objective was to assess Whether Voltage-gated calcium channel α_2_δ-1(Cavα2δ-1) is related to myofascial orofacial pain. Rats were divided into the masseter tendon ligation group and the sham group. Compared with the sham group, the mechanical pain threshold of the masseter tendon ligation group was reduced on the 4th, 7th, 10th and 14th day after operation(*P* < 0.05). On the 14th day after operation, Cavα2δ-1 mRNA expression levels in trigeminal ganglion (TG) and the trigeminal spinal subnucleus caudalis and C1-C2 spinal cervical dorsal horn (Vc/C_2_) of the masseter tendon ligation group were increased (*P*_*TG*_=0.021, *P*_Vc/C2_=0.012). Rats were divided into three groups. On the 4th day after ligating the superficial tendon of the left masseter muscle of the rats, 10 ul Cavα2δ-1 antisense oligonucleotide, 10 ul Cavα2δ-1 mismatched oligonucleotides and 10 ul normal saline was separately injected into the left masseter muscle of rats in Cavα2δ-1 antisense oligonucleotide group, Cavα2δ-1 mismatched oligonucleotides group and normal saline control group twice a day for 4 days. The mechanical pain threshold of the Cavα2δ-1 antisense oligonucleotides group was higher than Cavα2δ-1 mismatched oligonucleotides group on the 7th and 10th day after operation (*P* < 0.01). After PC12 cells were treated with lipopolysaccharide, Cavα2δ-1 mRNA expression level increased (*P* < 0.001). Cavα2δ-1 may be involved in the occurrence and development in myofascial orofacial pain.

## Introduction

Orofacial pain is a condition that affects the mineralized and soft tissue of the face and oral cavity [1]. Orofacial pain may be classified according to reported history and symptoms, including pain of musculoskeletal origin, dental pain, primary headache, neuropathy, neuralgia, etc [2]. Myofascial orofacial pain is the second most recurrent kind of orofacial pain, it is estimated that 33% of people have some symptoms in both the chewing muscles and face [3]. This condition is usually associated with temporomandibular joint dysfunction (TMD), which involves the periauricular area, chewing muscles, and related structures [4].

The International Classification of Orofacial Pain,1st edition (ICOP) divides myofascial orofacial pain into primary myofascial orofacial pain and secondary myofascial orofacial pain. Among them, Secondary myofascial orofacial pain includes myofascial orofacial pain attributed to tendonitis, myofascial orofacial pain attributed to myositis, myofascial orofacial pain attributed to muscle spasm [1]. It is generally assumed that myofascial orofacial pain related to TMD may originate from trigger points (TP), which are characterized by hypersensitivity of a taut band or palpable nodule and pain due to local muscle contraction, which can decrease the range of motion [3]. At present, the pathogenesis of myofascial orofacial pain is still unclear.

The treatment of myofascial orofacial pain is generally based on inactivating the TP [5]. There is no definite treatment method. And therefore, some potential methods have been used, including botox injections, passive stretching, ischemic compression, massage, transcutaneous electrostimulation nerve stimulation, infrared laser, biofeedback, ultrasound, and cognitive behavioral therapy [3, 6, 7].

Research models for myofascial orofacial pain include injection of glutamate, CFA, hypertonic saline, etc. into the masseter muscle, but these models maintain pain for only 1–3 weeks [8, 9]. Recently, it has been found that the myofascial orofacial pain model established by ligating the tendon of anterior superficial part of masseter muscle (TASM) can provide a long-lasting pain hypersensitivity and can better simulate the long-term existence of myofascial orofacial pain status [10, 11].

Calcium Channel α2δ-1(Cavα2δ-1) is a subunit of voltage-gated Ca^2 +^ channels (VGCCs) [12]. A large number of studies have shown that Cavα2δ-1 plays an important role in neuropathic pain. In models of neuropathic pain, the level of Cavα2δ-1 mRNA and protein on the injured side was significantly increased, and the increase in Cavα2δ-1 was related to the onset of allodynia [13]. Whether the change of Cavα2δ-1 expression is related to the development of myofascial orofacial pain is unclear.

Therefore, in this study, experiments of establishing myofascial orofacial pain model in vivo and experiments on PC12 neuron cell lines in vitro were used to clarify the role of Cavα2δ-1 in the occurrence and development in myofascial orofacial pain.

## Methods

### Animal experiment

#### Rearing rats

Thirty healthy 8-week-old male rats were selected (Beijing Weitong Lihua Technology Co., Ltd.) weighing 200–250 g and raised in the Laboratory of Animal Center of China Medical University. The feeding conditions were as follows: temperature (22 ± 2) ℃, relative humidity 40 -60%,12/12 h day and night alternating light, water and food ingested freely. The rats were acclimated in this environment for 3–5 days before the experiment. The experimental operation and animal feeding were in compliance with the basic regulations for animal experiments and animal ethics (2,018,067).

#### Establishment a myofascial orofacial pain model

Twelve rats were randomly divided into the masseter tendon ligation group and the sham group, with 6 rats in each group. The masseter tendon ligation group and the sham group were anesthetized with 2% pentobarbital sodium (50 mg/kg, intraperitoneal injection). Then the rats were fixed on the operating table to keep a head-up posture. In the masseter tendon ligation group, a 3 mm long anterior-posterior incision was made along the mucogingival junction of the first molar on the left side of the rats. TASM was separated carefully and gently. TASM was ligated by two chrome bowels (4.0), and the distance between them was 2 mm. Finally, the wound was sutured in layers, and apply an appropriate amount of antibiotics (gentamicin) to the wound. Rats in the sham group received the same operation except for ligation of TASM.

#### Behavioral testing

The rats of the masseter tendon ligation group and the sham group were placed in a quiet environment to keep the rats in a stable state. These rats were tested for mechanical pain threshold before operation and on the 4th,7th,10th and 14th days after operation. The von Frey filaments were applied vertically to the left masseter muscle area of the rats, and the mechanical pain threshold was tested. The positive reaction was to recede the body or dodge the head, scratch the face asymmetrically or bite the von Frey filaments. The von Frey filaments stimulation intensity started from 3.22 g, and when a positive reaction was caused, the von Frey filament of the adjacent lighter level was replaced. When a negative reaction was caused, the von Frey filament of the adjacent stronger level was replaced. The interval between each stimulation is 10 s, and the stimulation was repeated five times in total. The measurement was continued until the first negative and positive cross-reaction occurred. First the response frequencies to a series of von Frey filament forces [(number of responses/number of stimuli) *100%] were calculated, and then the stimulus-response frequency curve (S-R curve) was drawed. After a non-linear regression analysis, the EF50 value was obtained according to the S-R curve, which was defined as the effective von Frey filament force (g) that produced 50% of the response frequency. A decrease in EF50 indicated the presence of mechanical allodynia. The mechanical stimulus response threshold(EF50)was calculated by Up-Down Calculators.

#### Quantitative real-time polymerase chain reaction(qRT-PCR)

On the 14th day after operation, the rats of the masseter tendon ligation group and the sham group were anesthetized with 2% sodium pentobarbital (100 mg/kg), and the rats in both groups were sacrificed by decapitation. The head of the rat was fixed on a wooden board, the skull and brain tissue of the rat were exposed. The left trigeminal ganglion, trigeminal spinal subnucleus caudalis and C1-C2 spinal cervical dorsal horn (Vc /C_2_) were obtained. The total RNA was extracted in trigeminal ganglion and Vc/C_2_ tissue according to the instructions of Trizol reagent, and determine the concentration and purity of total RNA. cDNA was formed by reverse transcription using TaKaRa’s reverse transcription kit. The target cDNA was amplified using TaKaRa PCR kit. The PCR amplification conditions were: pre-denaturation at 95 ℃ for 30 s, 95 ℃ for 5 s, 61.4 ℃ for 30 s, 72 ℃ for 30 s, a total of 40 cycles, and 72 ℃ for 3 min.

The primer sequence was as follows:

Cavα2δ-1-forward:5’-TGAGTTGTTTCCAGCACCTG-3’;

Cavα2δ-1-reverse:5’-CTCTTCTCCTCCATCCGTGA-3’;

GAPDH-forward:5’-ACCACAGTC-CATGCCATCAC-3’;

GAPDH-reverse: 5’-TCCACCACCCT-GTTGCTGTA-3’;

Calculate the relative expression of the target gene using the 2^−ΔΔCt^ method. The mRNA expression levels of Cavα2δ-1 were compared in the masseter tendon ligation group and the sham group.

#### Antisense oligodeoxynucleotide treatment

Eighteen male rats were randomly divided into Cavα2δ-1 antisense oligonucleotides group, Cavα2δ-1 mismatched oligonucleotides group and normal saline control group, each with 6 rats. The antisense and mismatch oligonucleotides of Cavα2δ-1 were synthesized, which had 3 nucleotide phosphorothioate modifications at the 5’and 3’ ends respectively, which were purified by a high-purity salt-free method. Cavα2δ-1 antisense and mismatch oligonucleotides had 3 nucleotide phosphorothioate modifications at the 5’ and 3’ ends, respectively, and were purified with High Purity Salt Free method. The antisense oligonucleotide sequence of Cavα2δ-1 was AGCCATCTTCGCGATCGAAG, and the mismatch oligonucleotide sequence was CGATACCTCGCTGGCTAAAG. These sequences were dissolved in sterile saline. On the 4th day after the anterior superficial tendon of the left masseter muscle was ligated, the rats that injected Cavα2δ-1 antisense oligonucleotides into the left masseter muscle were defined as the Cavα2δ-1 antisense oligonucleotides group, 10 ul each time, twice a day for 4 days. The rats that injected Cavα2δ-1 mismatched oligonucleotides into the left masseter muscle were defined as the Cavα2δ-1 mismatched oligonucleotides group, 10 ul each time, twice a day for 4 days. The rats that injected normal saline into the left masseter muscle were defined as the normal saline control group,10 ul each time, twice a day for 4 days. The mechanical pain threshold of three groups was measured before operation, the 4th, 7th, 10th and 14th day after operation.

### Cell experiment

#### Cell culture

PC12 cells at passage 5 were placed in a DMEM high-glucose medium system containing 10% fetal bovine serum,100 U/mL penicillin and 100 ug/mL streptomycin, and cultured in a CO2 constant temperature incubator with a volume fraction of 5% at 37 ℃.After the cells grew to the logarithmic phase, the cell was digested to obtain a cell suspension, and the cell density was adjusted to 2 * 10^4^ cells/ml to inoculate a 6-well plate.

#### Lipopolysaccharides (LPS) treatment of PC12 cells

The control group: PC12 cells was cultured with the above-mentioned medium for 36 h.

The LPS treatment group: 24 h after PC12 cells were cultured with the above-mentioned medium, PC12 cells were treated with 5 ug/ml LPS for 12 h.

#### Quantitative real-time polymerase chain reaction(qRT-PCR)

The total RNA in the control group and the LPS treatment group was extracted according to the instructions of Trizol reagent, and the concentration and purity of total RNA were determined. cDNA was formed by reverse transcription using TaKaRa’s reverse transcription kit. The target cDNA was amplified using TaKaRa PCR kit. The PCR amplification conditions were: pre-denaturation at 95 ℃ for 30 s, 95 ℃ for 5 s, 61.4 ℃ for 30 s, 72 ℃ for 30 s, a total of 40 cycles, and 72 ℃ for 3 min.

The primer sequence was as follows:

Cavα2δ-1-forward:5’-TGAGTTGTTTCCAGCACCTG-3’;

Cavα2δ-1-reverse:5’-CTCTTCTCCTCCATCCGTGA-3’;

GAPDH-forward:5’-ACCACAGTC-CATGCCATCAC-3’;

GAPDH-reverse: 5’-TCCACCACCCT-GTTGCTGTA-3’;

Calculate the relative expression of the target gene using the 2^−ΔΔCt^ method. The mRNA expression levels of Cavα2δ-1 were compared in the control group and the LPS treatment group.

### Statistical analysis

The data was analyzed by Sigmaplot 14, SPSS 16.0 and Graphpad prism 5. The mechanical stimulus response threshold(EF50)was analyzed by a two-way repeated analysis of variance (ANOVA). Cavα2δ-1 mRNA levels were analyzed by two independent samples t test. *P* < 0.05 indicated that the difference was statistically significant.

## Results

### Experiment results of animal behavior

**Fig. 1:**
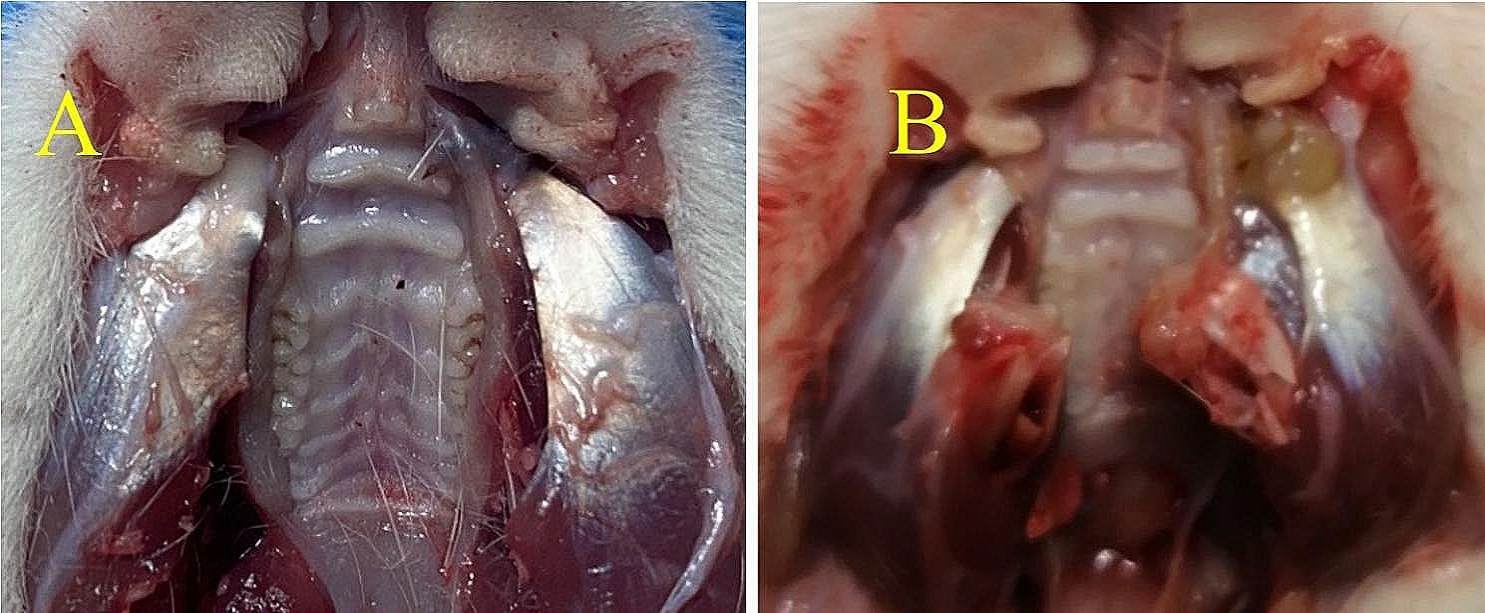
(**A**) the sham group (**B**) the masseter tendon ligation group

On the 14th day after operation, compared with the sham group, the masseter tendon on the ligated side of the masseter tendon ligation group changed significantly (Fig. 1.). The color of the tendon became dark yellow, indicating mechanical damage.

**Fig. 2:**
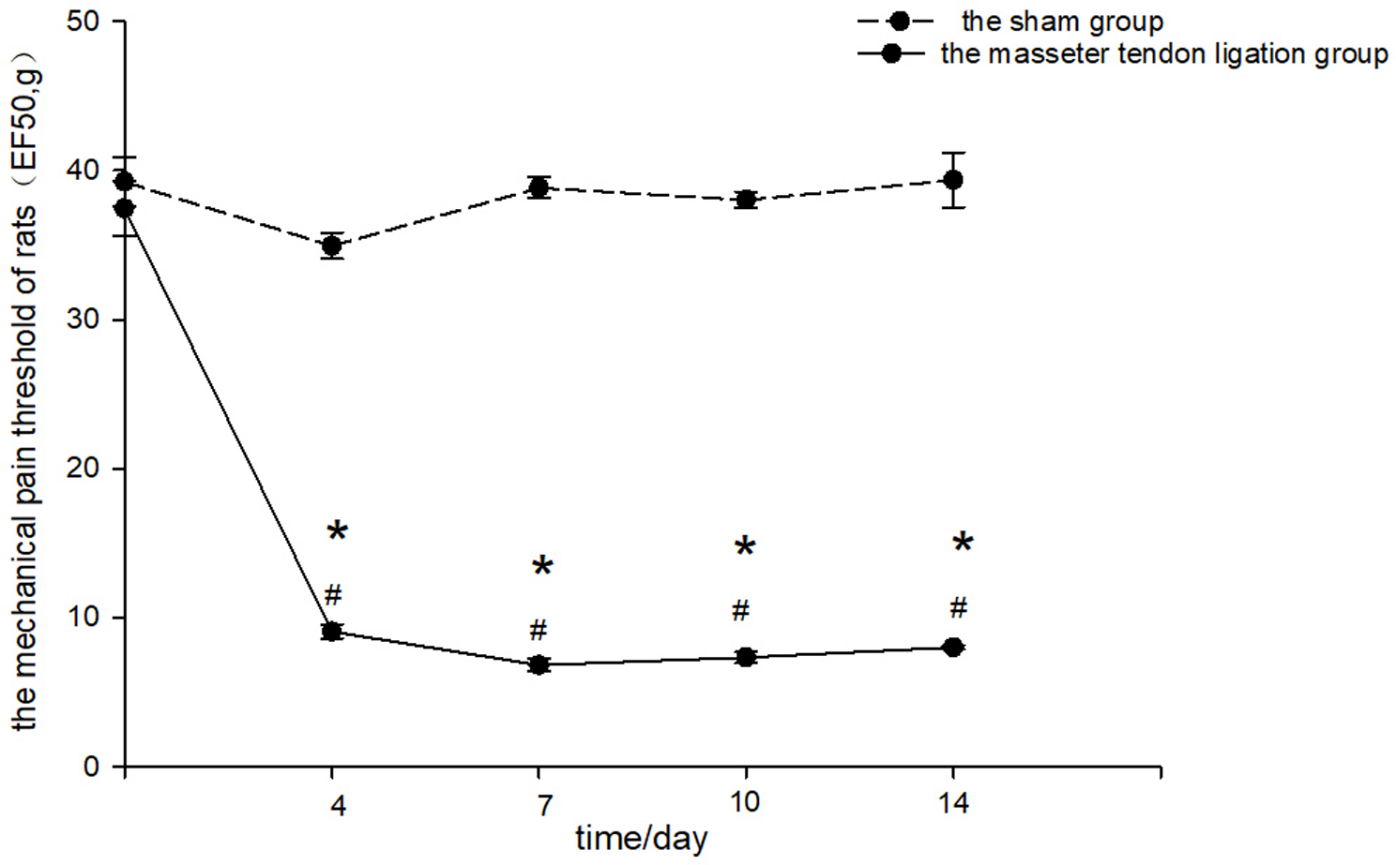
Mechanical pain threshold of the masseter tendon ligation group and the sham group. * vs. the sham group *P* < 0.05; # vs. before ligation *P* < 0.05

Compared with preoperatively, the mechanical pain threshold of rats in the masseter tendon ligation group was significantly reduced on the 4th, 7th, 10th and 14th days after operation(*P* < 0.001, Fig. 2.).Compared with preoperatively, the mechanical pain threshold of rats in the sham group did not change significantly on the 4th, 7th, 10th and 14th days after operation (*P* > 0.05, Fig. 2).The preoperative mechanical pain threshold of the masseter tendon ligation group was not significantly different from that of the sham group (*P* > 0.05, Fig. 2).The mechanical pain threshold of the masseter tendon ligation group was lower than that of the sham group on the 4th, 7th, 10th and 14th days after operation (*P* < 0.001, Fig. 2), confirming the successful establishment of the myofascial orofacial pain model.

### Cavα2δ-1 mRNA expression levels in trigeminal ganglion and Vc/C2 in rats of the masseter tendon ligation group and the sham group

**Fig. 3:**
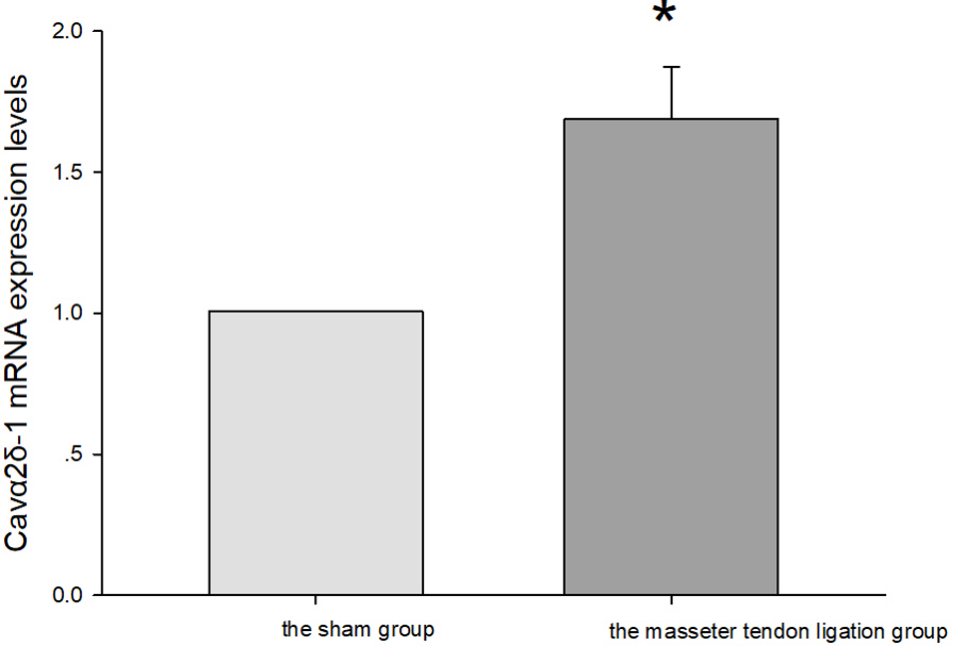
Cavα2δ-1 mRNA expression levels in trigeminal ganglion in the masseter tendon ligation group and the sham group. * vs. the sham group *P* = 0.021

Compared with the sham group, Cavα2δ-1 mRNA expression levels in the trigeminal ganglion of the masseter tendon ligation group were significantly increased on the 14th postoperative day (*P* = 0.021, Fig. 3.).

**Fig. 4:**
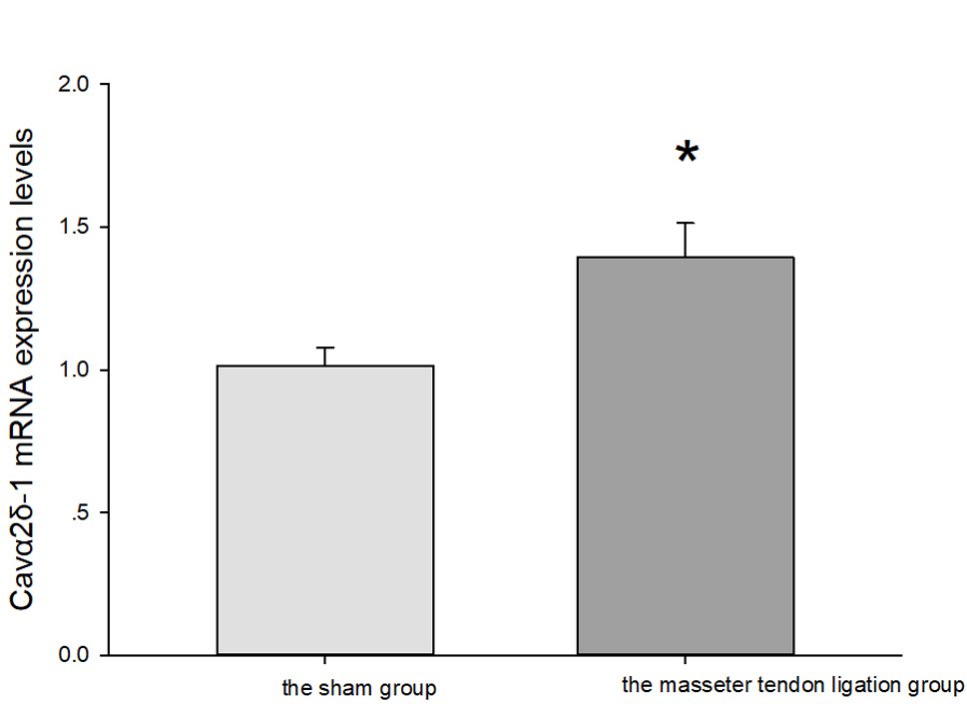
Cavα2δ-1 mRNA expression levels in Vc/C2 in the masseter tendon ligation group and the sham group. * vs. the sham group *P* = 0.012

Compared with the sham group, Cavα2δ-1 mRNA expression levels in the Vc/C2 tissue of the masseter tendon ligation group were significantly increased on the 14th postoperative day (*P* = 0.012, Fig. 4.).

### Behavioral experimental results of TASM rats after Cavα2δ-1 blockade treatment

**Fig. 5:**
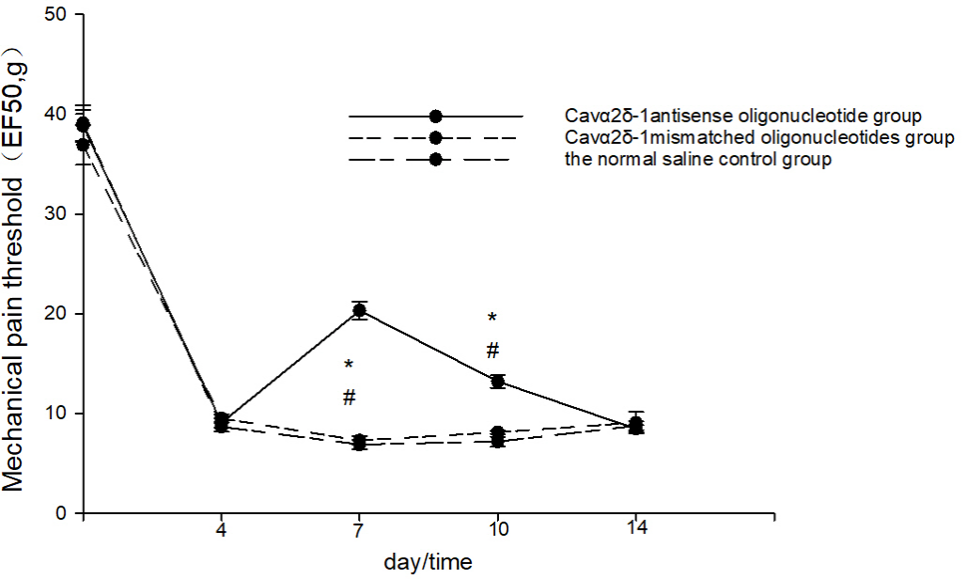
Mechanical pain threshold of Cavα2δ-1 antisense oligonucleotide group, Cavα2δ-1 mismatched oligonucleotides group and the normal saline control group. * vs. Cavα2δ-1 mismatched oligonucleotides group *P* < 0.05; # vs. the 4th day after the operation *P* < 0.05

Compared with the 4th day after the operation, the mechanical pain threshold of the rats in the Cavα2δ-1 antisense oligonucleotide group increased on the 7th and 10th days after the operation, and the difference was statistically significant (*P* < 0.001, Fig. 5.). The mechanical pain threshold of the Cavα2δ-1 antisense oligonucleotide group was higher than that of the Cavα2δ-1 mismatched oligonucleotide group on the 7th and 10th postoperative day, and the difference was statistically significant (*P* < 0.001, Fig. 5.). There was no significant difference in the preoperative and postoperative mechanical pain thresholds between the Cavα2δ-1 mismatch oligonucleotide group and the normal saline control group (*P* > 0.05, Fig. 5).

### Changes of Cavα2δ-1 expression levels after treatment of PC12 cells with LPS

**Fig. 6:**
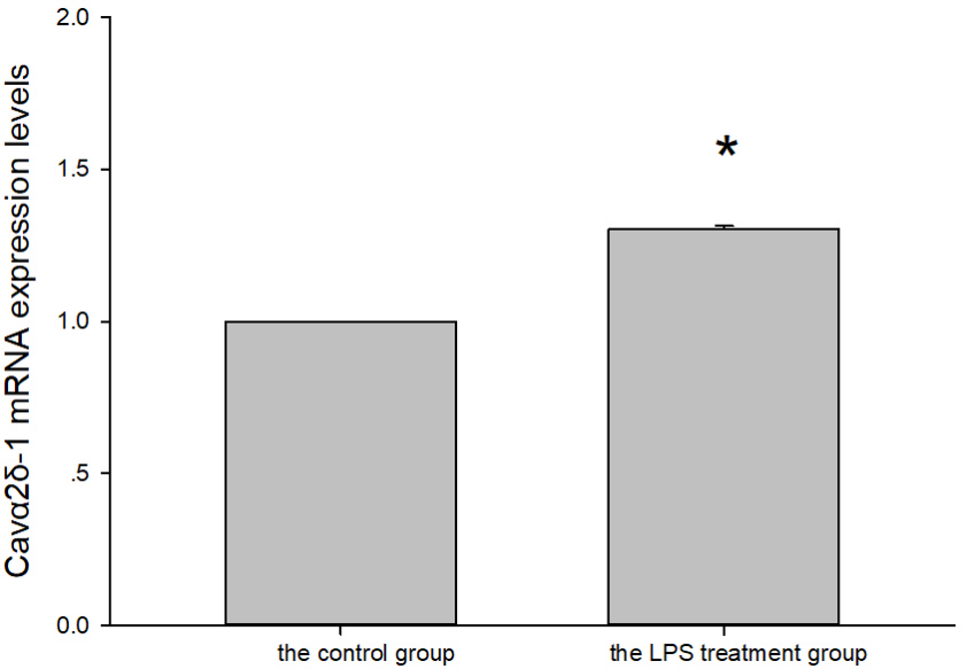
Cavα2δ-1 mRNA expression levels in the control group and the LPS treatment group. * vs. the control group *P* < 0.001

Compared with the control group, the expression levels of Cavα2δ-1 mRNA in the LPS treatment group were significantly increased (*P* < 0.001, Fig. 6.).

## Discussion

Orofacial pain and headaches are highly prevalent diseases but are usually difficult to treat. Headaches are classified as migraine, tension-type, trigeminal autonomic cephalgia (e.g., cluster headache), etc. Headaches or other facial pain have a larger differential [2]. It was supported by neurophysiological studies that a dysfunction of pain control systems in a role of the brainstem and headaches in their pathogenesis. Abnormal sensitization, lower cortical pre-activation and loss of habituation were seen in migraine. Decreased pain thresholds and altered pain perception were found In cluster headaches (CH) [14, 15]. A CH attack lasts between 15 and 180 min, and multiple attacks per day may occur, whereas the duration of a migraine attack is between 4 and 72 h, and recurrence is defined as a headache within 22 h of initial successful treatment of a migraine attack (2-hour headache response) [16].

Myofascial orofacial pain is one of the highest incidence rates and the most intricate kinds of orofacial chronic pain to treat [9]. This study established a myofascial orofacial pain model by ligating the anterior superficial masseter tendon, which is unique in that it provides a durable pain hypersensitivity response that better mimics myofascial orofacial pain. In this study, the mechanical pain threshold of the masseter tendon ligation group was significantly lower than that of the sham operation group, confirming the successful establishment of the myofascial orofacial pain model.

The trigeminal ganglion is the first-order neuron of orofacial pain conduction, and Vc /C_2_ is the second-order neurons of orofacial pain conduction [17, 18]. In rats with myofascial orofacial pain, Cavα2δ-1 mRNA expression levels were elevated in the trigeminal ganglion and Vc /C_2_ on the injured side. After treatment with Cavα2δ-1 antisense oligonucleotide, the pain of myofascial orofacial pain was relieved, suggesting that Cavα2δ-1 may be involved in the development of orofacial myofascial pain.

Cavα2δ-1 is a subunit of voltage-gated Ca^2 +^ channels (VGCCs). VGCCs mediate the release of hormones and neurotransmitters, membrane excitability, synaptic and neuronal structural plasticity, muscle contraction. Cavα2δ-1 is widely distributed in the central and peripheral nervous systems, skeletal muscle, smooth muscle, cardiac muscle and endocrine tissues [19, 20].

Recent literatures indicate that Cavα2δ-1 is related to the occurrence and development of neuropathic pain. In spinal nerve injury, the expression level of Cavα2δ-1 on the injured side is up-regulated, and the upregulation of Cavα2δ-1 is related to neuropathic pain. Transgenic mice overexpressing Cavα2δ-1 have lower pain threshold, while intramedullary injection of Cavα2δ-1 inhibitory ligand gabapentin can significantly reduce pain sensation [21, 22]. The results of this study showed that in the myofascial orofacial pain model, Cavα2δ-1 mRNA expression levels were elevated in trigeminal ganglion and Vc /C_2_ on the injured side. After Cavα2δ-1 antisense oligonucleotide treatment, the pain degree of myofascial orofacial pain was reduced, which was consistent with the role of Cavα2δ-1 in neuropathic pain reported in the literatures. However, the specific mechanism of Cavα2δ-1 in the development of myofascial orofacial pain remains unclear.

Cavα2δ-1 is coupled to N-methyl-d-aspartate receptor (NMDAR) and mediates neuropathic pain caused by nerve injury [23]. Overexpression of Cavα2δ-1 can enhance the activity of presynaptic and postsynaptic NMDAR in spinal dorsal horn neurons, which can cause pain hypersensitivity. When Cavα2δ-1 is ablated or knocked down, the synaptic NMDAR activity increased by nerve injury becomes normalized. Cavα2δ-1 directly interacts with NMDAR through its C-terminal, increasing pre-synaptic NMDAR activity. The enhanced pre-synaptic NMDAR activity increases the release of glutamate from the primary afferent end to the spinal dorsal horn neurons, which promotes the development of neuropathic pain [24–26]. In addition, gabapentinoids reduce pain hypersensitivity by acting on α2δ-1–bound NMDARs ^[16]^.

Cavα2δ-1 interacts with thrombospondin 4 and also contributes to the development of neuropathic pain. Thrombospondin 4 is a class of oligomeric extracellular matrix glycoproteins [27]. In the spinal cord injury pain model, thrombospondin 4 interacts with Cavα2δ-1 to promote dysexcitatory synaptogenesis and is involved in pain. Blockade or downregulation of Cavα2δ-1 blocked thrombospondin 4-induced pain in in vivo experiments in sciatic nerve-ligated mice and in vitro in neuronal cell cultures. Blockade or knockout of thrombospondin 4 blocked behavioral hypersensitivity induced by overexpression of Cavα2δ-1. Thrombospondin 4 regulates Cavα2δ-1 through T-type calcium channels, which induces increased frequency and amplitude of excitatory postsynaptic currents and increased expression of the presynaptic marker VGlut2 and the postsynaptic marker PSD95. Thrombospondin 4 acts only on newly created synapses. Therefore, gabapentin, early use of the ligand of Cavα2δ-1, can block thrombospondin 4-induced synaptogenesis and neuropathic pain [28, 29]. In dorsal root ganglia, Thrombospondin 4 promotes peripheral sensory system hypersensitivity by reducing HVA and increasing LVA in DRG neurons through T-type calcium channels [30]. In Cavα2δ-1-overexpressing transgenic mice, EGF-LIKE domain-induced pain-induced in vivo experiments in rats, and in vitro experiments in retinal ganglion cells (RGC), Thrombospondin 4 may interact with the von Willebrand factor A (VWA) domain of Cavα2δ-1 through its EGF-LIKE domain to reduce HVA ICa in primary neurons and increase LVA ICa, which can promote the formation of excitatory synapses in sensory neurons and hypersensitivity reactions [31–33].

The factors leading to constant pain after TASM ligation involve cellular changes at the site of injury, such as mechanical breakdown of collagen fibers, inflammatory responses, damage of peripheral nociceptors, etc [11]. LPS is a component of the cell wall of Gram-negative bacteria and is a potent inducer of inflammatory responses [34, 35]. PC12 cells were derived from rat pheochromocytoma. Rat Pheochromocytoma is a Gangliocytoma [36]. Endotoxin LPS can induce a normal animal immune system response. In vitro models of lipopolysaccharide-induced PC12 cells are commonly used for inflammation studies [37]. In the present study, the expression level of Cavα2δ-1 mRNA was increased in LPS treatment group.

In this study, a model of myofascial orofacial pain was established by ligating the anterior superficial tendon of the unilateral masseter muscle, and the role of Cavα2δ-1 in the occurrence and development of orofacial myofascial pain was innovatively studied, to provide an experimental basis for the treatment of myofascial orofacial pain.

### Limitations of this study

However, this study does have certain limitations. The study was for a short interval, and further studies are demanded to evaluate its long-term effect. And the detailed mechanism of Cavα2δ-1 involved in myofascial orofacial pain requires further investigation and will be explored in future research, to provide an experimental basis for the treatment of myofascial orofacial pain.

### Conclusion

Cavα2δ-1 may be involved in the occurrence and development in myofascial orofacial pain.

## Data Availability

The datasets used and/or analysed during the current study available from the corresponding author on reasonable request.
